# Data sharing ethics toolkit: The Human Cell Atlas

**DOI:** 10.1038/s41467-024-54300-3

**Published:** 2024-11-20

**Authors:** Emily Kirby, Alexander Bernier, Roderic Guigó, Barbara Wold, Fabiana Arzuaga, Mayumi Kusunose, Ma’n Zawati, Bartha M. Knoppers

**Affiliations:** 1 Centre of Genomics and Policy, School of Biomedical Sciences, Faculty of Medicine and Health Sciences, McGill University, 740 Dr. Penfield, Suite 5200, Montreal, QC Canada; 2 Bioinformatics and Genomics, Center for Genomic Regulation (CRG), The Barcelona Institute for Science and Technology (BIST), Dr. Aiguader 88, Barcelona, Catalonia Spain; 3 Universitat Pompeu Fabra (UPF), Barcelona, Catalonia Spain; 4 Division of Biology and Biological Engineering, California Institute of Technology, Pasadena, CA USA; 5 Interministerial Comission on Advanced Therapies Ministry of Science, Technology and Innovation -Argentina Godoy Cruz 2320. 4th Floor, Ciudad Autónoma de, Buenos Aires Argentina; 6 Center for Integrative Medical Sciences, RIKEN. 1-7-22 Suehiro-cho, Tsurumi-ku, Yokohama City, Kanagawa Japan

**Keywords:** Ethics, Genetic databases, Databases, Law

## Abstract

Striving to build an exhaustive guidebook of the types and properties of human cells, the Human Cell Atlas’ (HCA) success relies on the sampling of diverse populations, developmental stages, and tissue types. Its open science philosophy preconizes the rapid, seamless sharing of data – as openly as possible. In light of the scope and ambition of such an international initiative, the HCA Ethics Working Group (EWG) has been working to build a solid foundation to address the complexities of data collection and sharing as part of Atlas development. Indeed, a particular challenge of the HCA is the diversity of sampling scenarios (e.g., living participants, deceased donors, pediatric populations, culturally diverse backgrounds, tissues from various developmental stages, etc.), and associated ethical and legal norms, which vary across countries contributing to the effort. Hence, to the extent possible, the EWG set out to provide harmonised, international and interoperable policies and tools, to guide its research community. This paper provides a high-level overview of the types of challenges and approaches proposed by the EWG.

## Abstract (web summary)

The human cell atlas (HCA) is intended as an exhaustive guidebook of human cell types and their properties. Here Kirby et al. outline how the HCA Ethics Working Group is working to build a solid foundation to address the complexities of data collection and sharing.

## Introduction

In recent years, members of the international research community have mobilised to enable streamlined, international data sharing, particularly in the context of open science initiatives. Open science is broadly defined as a movement which seeks to leverage new practices and digital technologies to increase transparency and access in scholarly research^1^, and improve reproducibility and replicability of research findings^2^. Data sharing and access to resources (data, methods, publications) are important pillars of the open science movement, and both aim to ensure rapid and equitable access to pre-competitive, raw research resources^3^.

Following the rise of data-driven, infrastructure research and global referencing initiatives such as the Human Genome Project^4^, the International HapMap Project^5^, the 1000 Genomes Project^6^, the Human Pangenome Project^7^ and the International Cancer Genome Consortium^8^, issues such as benefit-sharing, access to data and inclusive participation, have been fundamental considerations of research consortia. Large volumes of data are required to statistically power analyses of common and rare diseases, often requiring important data storage and analytical capacities (e.g. cloud computing, centralized or federated platforms)^9^. Biomedical research shows promise because this volume of data can now be generated.

The Human Cell Atlas (HCA) is an ambitious project hoping to uphold principles underlying open science and data sharing. Its goal is to map gene expression and other molecular profiles of all cell types and cell states, tissues, organs, and organ systems in the ‘healthy’ human body. The construction of this reference map will then enable research into dysregulated cell states contributing to disease and leading, eventually, to the identification of biomarkers and potential therapeutic targets^10,11^. The impact of the initiative relies both on diversity (donor/participant demographic and phenotypic characteristics, tissue types, developmental stages, participating regions/countries, etc.) and on the collaboration of scientists, research donors/participants, and communities, in order to obtain the number of cells and datasets required to understand what a reference cell type and a cell state might look like^12^.

The HCA proposes to build a research infrastructure. It is not a research project in itself as it does not directly recruit participants, sample or analyse tissues. Rather, building the Atlas relies on the contribution of scientists around the world, who agree to contribute data from their own local research projects to this combined effort. As part of the efforts, an Ethics Working Group (EWG) was created early in the inception of the HCA. Given the scope of the initiative, the EWG did not set out to weigh in or resolve all ethical and legal issues that may be encountered by the HCA consortium or its members, nor did it provide an exhaustive review of all normative frameworks governing the contribution of data in all jurisdictions or countries involved. Rather, the focus of the EWG was to assess the structural issues pertaining to contribution to the atlas from pre-existing projects and data sharing in the context of an international collaborative effort.

The composition of the EWG was carefully selected to encompass approximately 18 members, including lawyers, ethicists, genomic scientists, representatives of the main regional networks of the HCA and knowledgeable on the breath of ethical issues expected to arise (e.g., data protection/privacy, human tissue sampling, deceased donors, data sharing, developmental biology, paediatrics, open science and IP, etc.). The core membership is supported by a dozen active observers of the Working Group, whose role is to provide input on key scientific, technical, and strategic funding initiatives and needs of the HCA. In addition, the work of the EWG is also informed by other activities of the HCA, including the work of the Equity Working Group, which specifically works to engage the global community spanning diverse geographic and ethnic groups in order to drive inclusive representation and participation and promote equal benefit from the HCA.

This paper provides a high-level overview of the principal ethical and legal elements discussed by the EWG, in relation to data sharing and the ethical governance of infrastructure science. It then describes the tools and resources created by the EWG to assist the research community in sampling cell tissues and contributing datasets to this large-scale, collaborative effort. Finally, we propose some lessons learned in the development of tools and guidance for international collaborative health research, in the context of a fragmented international normative ecosystem. The objective is to provide insight into the role of advisory groups, such as the EWG, within international collaborative research. These groups often operate outside the realm of systematic research but provide critical and timely resources to the set-up and functioning of such initiatives.

A lay summary of this article in English and in other languages can be found at: https://zenodo.org/communities/hcaewg/.

### Overview of normative framework and ethical issues

Given the large-scale, international and collaborative nature of the HCA, the EWG first set out to identify areas of tension between existing normative frameworks. Then, alongside the development of data governance within the consortium, it developed tools to enable widespread participation by scientists around the world in the creation of the Atlas.

Biomedical research, especially -omics research, is governed by a complex framework of policies, guidelines, laws, regulations and best practices (together, these rules are referred to as a ‘normative framework’). Historically, international ethical policies and guidelines, such as the World Medical Association’s (WMA) *Declaration of Helsinki* (2024), the Council for International Organisations of Medical Sciences (CIOMS) *International Ethical Guidelines for Health-related Research Involving Humans* (2017), and the United Nations Educational, Scientific, and Cultural Organisation, Bioethics Programme’s (UNESCO) *Universal Declaration on Bioethics and Human Rights* (2005), have proposed foundational principles for ethical conduct of research with humans. Core ethical issues addressed across guidelines include, for instance, the protection of human dignity, integrity, right to self-determination (autonomy), privacy, and confidentiality of personal information, which are implemented through practices such as evaluating the risks and benefits of research, free and informed consent, evaluation of research by an ethics committee, and measures to protect vulnerable populations.

More recently, a number of international policy documents have built on the existing normative framework to account for the increasing research collection, use and sharing of data derived from human research, including health-related phenotypic data and -omics data. These include, for instance, the WMA’s *Declaration of Taipei* (2016), the Global Alliance for Genomics and Health’s (GA4GH*) Framework for Responsible Genomic and Health-related Data* (2014), the Organisation for Economic Co-operation and Development (OECD)’s *Recommendation of the Council on Human Biobanks and Genetic Research Databases* (2009) and *Recommendation of the Council on Health Data Governance* (2017), as well as the UNESCO’s *Recommendation on Open Science* (2021). While these guidelines reaffirm the fundamental principles underlying research ethics, a few key adaptions have been made in relation to the particular considerations surrounding data governance, use and sharing, including the creation of health databases, data management, privacy considerations, and future/secondary uses of data, etc.

The HCA is distinctive from a research ethics perspective as the applicable normative ecosystem governing data contributors and users calls upon both guidelines related to foundational research ethics, as well as more recent norms pertaining to the sharing of health-related and -omic data. The EWG examined a number of topics at the intersection of these norms.

### International collaborative research & research ethics oversight

First, as an international, decentralised collaborative initiative, the HCA evolves in a distinct ecosystem of ethical oversight. Many international bioethical norms approach collaborative research using a ‘multi-centric research’ model, whereby a common protocol is reviewed by ethics committees at each participating site, who then evaluate the project based on local requirements. However, the contribution of data to the HCA is ensured through different research projects in different countries, each with their own protocol. The HCA, therefore, has little control over the ethical approval of contributing projects and must rely on a patchwork of local approvals. This decentralised model, while allowing for consideration of local cultural, ethical or regulatory specificities by ethics committees, can be difficult to navigate when building data-intensive infrastructures. In particular, it has been noted that local committees after often ill-equipped to evaluate data sharing and its implications, particularly as part of larger initiatives^13–15^.

To account for this complexity, the EWG developed tools for guidance and support to contributors to the HCA and their local ethics committees (see Table 1). A key objective was to streamline local ethical reviews, and strike a balance between providing common ethical elements within the HCA (e.g., core consent elements, information on data governance/access, retrospective consent assessment tools, etc.), while allowing local committees to independently assess any additional considerations applicable in their own jurisdiction or context (e.g., research with vulnerable groups or populations, regulatory requirement or models pertaining to tissue sampling, data protection laws, etc.).

**Table 1:** Overview of the categories of tools developed for the HCA ethics toolkit. The full toolkit is available online at: humancellatlas.org/ethics

Category	Description/purpose
General Ethics and Data Governance	Explain the HCA project in simple terms, providing useful background documents for institutional ethics review committees tasked with approving the collection of tissues and the contribution of data to the HCA
Consent tools	Templates and assessment tools for different HCA sampling scenarios (adult participants; addendum to consent forms for sampling of clinical leftover tissues; consent template for deceased donors; templates for the collection of developmental tissue samples; consent filter assessment tool for legacy datasets) A paediatric portfolio is available alongside the main consent tools to address certain issues specific to the paediatric cell atlas, including templates for the assent of minors as well as the consent of mature minors/parents/legally authorised representatives.
Data submission and sharing between sites	Template Material/Data Transfer Agreement, which incorporates key elements specific to the HCA, particularly with respect to open data sharing. Policies for the implementation of a managed access tier for certain datasets. Implementation of a Data Access Compliance Office (DACO) and Data Access Committee (DAC) (including implementation procedures)
Additional tools	Background reading material on the following topics: ‘Building the Human Cell Atlas: Issues with Tissues’^17^, ‘Children’s Right to Health’^50^, and ‘Children’s Data Protection’^51^
Ethics Helpdesk	Helpdesk for the HCA community to submit questions and to ask for certain topics to be brought to the attention of the HCA EWG

### Sampling human tissue

Second, to create a map of the human body, different tissue sampling scenarios must be envisaged. For instance, to understand how cells change and evolve throughout life, the entire developmental lifespan must be adequately captured, from gametes, embryos, and fetuses to children and adults – both living and deceased (some tissues cannot be sampled from living donors)^16^. Even within a single jurisdiction, these different types of tissue sampling populations call upon the application of different sets of rules or policies^17^, including norms related to consent, tissue acquisition, data/sample sharing or data protection considerations^12,16,17^. This presents an important, but not insurmountable, challenge to global projects such as the HCA, particularly in relation to acquiring tissues at different life stages.

As part of early background normative research, the EWG developed thematic ‘primers’ to identify and examine local practices related to consent and data sharing across a number of different jurisdictions representative of HCA regions^17^. As anticipated, this work concluded that the way in which the interests of research participants or tissue donors are protected, as well as their level of protection, are culturally specific and vary across countries^17^. As an example, while the universal requirement of free, informed consent ensures that research respects individual autonomy and choice, how this essential requirement is satisfied varies across jurisdictions and across tissue acquisition scenarios. Examples of this variation include the recognition (or not) of models such as: “opt-in”; free, informed consent; consent to secondary uses; broad consent; presumed consent (opt-out); or waivers^17^.

A challenge for the HCA EWG was, therefore, to identify the type of tools (e.g., consent form models, guidance for recruiters) needed to guide data contributors in relation to the complex task of assembling an ‘interoperable’ global, dataset, while recognising and respecting the wide range of ethical provenance related to tissue acquisition. Practically speaking, in some instances, this meant providing variations of the same template or document, to account for different regional models (as an example, post-mortem tissue donation is considered a ‘gift’ in certain jurisdictions, while in others, it remains human research requiring research consent– and therefore, different templates were proposed).

### Data protection and privacy considerations

Third, data protection and privacy considerations related to genomic data, particularly in relation to technological innovations, are rapidly evolving. As mentioned, not only have research ethics norms been adopted specifically for the sharing and use of health data, but the increased global scrutiny surrounding the protection of personal data has also had an important impact on data sharing for research purposes.

**Fig. 1:**
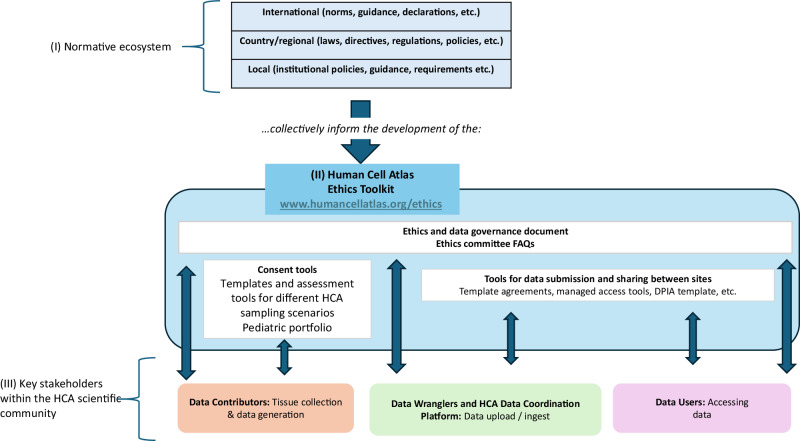
The ecosystem underlying the construction of the HCA ethics toolkit and its key HCA community stakeholders. This figure provides an overview of the ecosystem underlying the construction of the HCA ethics toolkit and its key HCA community stakeholders. **(I) Normative Ecosystem**. The top of the figure depicts the sources of policies, norms and regulations that inform how tissues and data can be collected and shared, for contribution to international efforts such as the HCA. **(II) Ethics Toolkit**. In turn, these rules informed the development of different tools proposed in the HCA ethics toolkit which are publicly available at humancellatlas.org/ethics (blue rectangle). Different tools proposed are intended for different stakeholders of the HCA community. For instance, the ‘Ethics and data governance document’ and the accompanying ‘Ethics committee FAQs’ are intended to provide an overall description of the HCA, its data platforms and data release model in language that is accessible to data contributors, and their local ethics committees. These documents are also relevant to other HCA stakeholders as they have been developed by engaging different groups, including platform developers, data wranglers, and potential users (as depicted by the blue arrows). Consent tools are mainly intended for contributors to the HCA (as depicted by the blue arrows). The EWG engaged with specific contributor communities for sector-specific guidance, for instance, in the areas of paediatrics, developmental tissues, or clinical sampling. Finally, tools related to submission and sharing between sites are of particular use to both data wranglers as well as data users (as depicted by the blue arrows). **(III) Key Stakeholders**. As the atlas is developed, we will continue to pilot different tools (e.g., data sharing and contribution agreement templates) through engagement with these stakeholders within the HCA community, and monitor whether adjustments are needed.

In this context, an important question for the HCA was to assess whether data incorporated in the atlas and subsequently shared (including phenotypic metadata as well as data derived from single cell sequencing technologies), constitutes personal data, as defined under several data protection regimes. This determination would then have important repercussions for the different stakeholders of the consortia, given their role as potential data processors^12^. Although this discussion was initially instigated by the entry into force of the General Data Protection Regulation^18^, it rapidly became apparent that this analysis would be relevant to other jurisdictions with similar protection regimes for data considered personal or identifiable. Ultimately, and similarly to other health research consortia^19^, a pragmatic approach was adopted whereby an ongoing assessment of data types (e.g., gene count matrices, sequencing data, metadata) and proportionate governance models were implemented (e.g., controlled or managed access)^12^ while proposing a release of appropriately consented data in open access when possible. This approach was then described in the Ethics and data governance document proposed in the toolkit (Table 1 and Fig. 1), to convey the model to bodies such as research ethics committees and institutional representatives, ultimately authorising data contribution.

### Open science, data sharing, access and use

Fourth, data sharing and open science have increasingly been discussed in the context of infrastructure science. The concept of ‘open science’ has been widely used to refer to several components of the research endeavour – including intellectual property, publication, and access to resources (such as data, protocols, tools and methods). Sectoral guidelines such as the FAIR^20^ and CARE^21^ principles and the GA4GH *Framework for Responsible Sharing of Genomic and Health-related Data*^22^, have proposed frameworks to foster data discovery, accessibility, interoperability and re-use. While the HCA’s activities relate to several facets of open science, the HCA EWG was more specifically involved in examining open science in the context of data sharing.

On the one hand, open data sharing strives to enable unencumbered, rapid access to greater sample sizes, fostering large-scale analyses, replicability and transparency^23,24^. Advocates of open science have emphasised the potential financial impact of openly sharing data resources through broader access, particularly for researchers and institutions in low-income settings and citizen scientists. On the other hand, if not adequately implemented, open data sharing can fail in reaching its objectives and, in some cases can cause unintended harm. For instance, there are privacy concerns with sharing some types of data publicly (even if permissible under data protection laws and appropriately consented). Furthermore, because there is less control and visibility of how the data is being accessed and used, there is a potential for use that could be considered inappropriate and could result in harm to groups/communities (e.g., stigmatisation)^23^, individuals (e.g., linkage with external datasets, re-identification attempts, discrimination), or society (e.g., data scraping/harvesting and use of artificial intelligence algorithms for future unknown analyses, etc.).

In the case of the HCA, it also became apparent that imposing an ‘all or nothing’ approach to open data sharing might actually impede researchers from certain regions or groups from contributing data to the atlas, particularly where more conservative regulatory regimes exist, or where cultural, ethical norms or donor consents limit public sharing of data^12^. This tension is also apparent in frameworks where data sovereignty and authority over data is central to equitable participation, for instance, in the context of sharing data from Indigenous Communities^25,26^. Ultimately, inappropriate implementation of open science could lead to inequities in the representativity of the atlas and diminish its scientific value and collective benefit.

In practice, data sharing proposes a spectrum ranging from controlled (or managed) access data to open (or public) data. Given that the HCA strives to uphold open science principles, ongoing discussions between a number of HCA stakeholders (including data contributors, the HCA Data Coordination Platform, the EWG and the Organising Committee) were centred around adopting processes and infrastructure to enable as much sharing of open (public) data as possible while recognising the need to protect and govern certain datasets under managed access. Again, through discussions with the HCA community, these decisions and processes were translated into various documents of the ethics toolkit (such as the Ethics and data governance and consent templates) so as to replicate and disseminate this information throughout the data lifecycle (consent, contribution, storage, sharing).

### Building the HCA ethics toolkit

To develop a common approach to data sharing within the consortium^22^ and to navigate the ethical and legal landscape described above, the EWG strived to develop practical guidance and tools (Table 1). These needs were addressed throughout the research data lifecycle, engaging with potential HCA scientific stakeholders in the process– from recruitment/collection of tissue samples to storage and analysis within the HCA Data Coordination Platform (DCP), and fsh/sharing (see Fig. 1).

For data contributors and their institutions, guidance was needed around consent language and providing explanatory materials for tissue sampling sites to enable contribution to the atlas (particularly with respect to open access data). Streamlined, high-level explanations regarding the structure and governance of the HCA were also needed to guide local ethics committees in their understanding of the initiative. The HCA consortium itself, particularly the DCP, called upon the EWG to provide input on data protection matters and data sharing within an open science context. Liaison with other similar data-sharing initiatives with existing ethical frameworks – including specific projects (e.g., GTEx) and standards organisations (Global Alliance for Genomics and Health (GA4GH)) – ensured that the tools developed for the HCA remain anchored into the existing data and policy ecosystem. Indeed, the EWG must work towards the goal of interoperability between existing and future initiatives, in the spirit of fostering (re)use of datasets^15,22,27^.

Proposing practical tools to address the ethical needs of the HCA research community was an important step to fostering pragmatic, actionable approaches to data sharing within the Atlas. While this article can only provide a high-level overview of the different facets of the ethics toolkit, we invite readers to learn more about the underlying approach proposed for each tool by consulting the publicly accessible resources available at: www.humancellatlas.org/ethics.

The ‘-omics’ research community has long been advocating for the development of tools and standards to harmonise approaches to data sharing^3,15,28–31^. Organisations such as the GA4GH work to enable responsible sharing of clinical and genomic data through both policies and harmonised data aggregation and federated approaches, to advance genomics medicine and research^29^. The HCA is a driver project of the GA4GH, meaning that it is an initiative that shapes GA4GH products and applies them to real genomic data^32^. The tools in the HCA Ethics Toolkit were inspired by both the GA4GH Framework for Responsible Sharing of Genomic and Health-related Data^22^ and by policies developed by the GA4GH Regulatory and Ethics Work Stream (REWS), in an effort to harmonise HCA practices with other global -omic initiatives^22,33–37^.

### Lessons learned: building large-scale research infrastructures in a fragmented normative environment

Developing actionable guidance for researchers to contribute to large-scale initiatives can encourage participation across contexts and regions despite differences in normative frameworks. We argue that these ethics and policy tools are representative of broader socio-cultural and policy issues that a number of international data-sharing research consortia must contend with refs. ^38–41^. Learning from the HCA EWG experience, we propose that the development of ethics and policy tools for collaborative data sharing should attempt to foster: (1) interoperability, (2) scalability/flexibility and (3) actionability.

In the field of biomedical research, interoperability is generally defined as enabling the meaningful comparison and combination of data across different research efforts or research sites^42^. Achieving interoperability (or a certain degree of harmonisation), is essential to the scientific endeavour, particularly in the context of international collaborative research consortia, which ultimately rely on the contribution of members (and member projects)^35^. Ethical and legal rules, standards or policies are a potential source of heterogeneity in datasets^30,42^ and can limit or place conditions on the contribution of data to the consortium as well as on further data sharing by the consortium. Interoperability can therefore be achieved by working on the architecture of the consortium upstream of data contribution (for example, by making template consent language, available to contributors, by proposing different models based on known regional variations), to ensure that entering datasets are suitable for further sharing as envisaged.

As an example, a review across seven countries of approaches to consent to tissue sampling for research use, undertaken as part of HCA EWG work, found that while there was a universal requirement of free, informed consent, the implementation of this essential requirement varies in different countries (the traditional “opt-in” model to free, informed consent, as compared to consent to secondary uses, broad consent, presumed consent (opt-out), and waivers)^17^. Moreover, even where there are high-level commonalities between jurisdictions, key differences remain that are specific to types of tissues and of research (for instance, embryonic/foetal tissue, paediatric or adult)^17^, particularly in more culturally sensitive research areas (e.g., developmental biology). Given this diverse context, we identified key thematic areas where normative commonalities could be identified, particularly in light of international guidelines.

In the context of the HCA, interoperability efforts were integrated in a number of consent tools designed for data contributors. For retrospective datasets, which include datasets generated from legacy tissue samples (e.g., archival samples, samples collected for a purpose other than research, etc.) and datasets that were generated before the creation of the HCA, an assessment tool was developed for contributors to determine whether datasets are suitable for inclusion in the HCA. This assessment is based, amongst others, on the source of the tissue sample, the donor consent to broad international data sharing, the donor consent for open data sharing, and/or whether reconsent/waiver of consent is feasible^43^. For prospective datasets - meaning datasets to be specifically consented for use in the HCA - core consent elements^44^ were proposed. These minimal clauses should explain particularities surrounding the generation of research data from tissue samples, international sharing, future use, commercial use, public (open) access, storage on cloud servers, duration of storage, data withdrawal and re-identification.

In addition to fostering interoperable datasets, the ethics toolkit was developed as a scalable model, meaning that it can be adapted to use across different data contribution and access scenarios. Because of the vast differences in normative frameworks in the different jurisdictions likely to contribute data to the HCA, it was difficult for the EWG to impose a single approach (all-or-none open data) without trading off the scientific value of the data collection. The EWG, therefore, did not impose normative decisions but rather, worked to create adaptable tools that could accommodate as many options as possible (e.g., different tissue sampling sources, consent models, etc.). A limitation of scalability, however, could be that contributors eventually deviate from the models proposed (for example, in adapting their own local consent forms). Further refinement and revision of the current tools proposed could be envisaged through additional consultation with stakeholders and toolkit users, by compiling and analysing changes made to existing templates once the first version of the toolkit has been in use for a certain period of time.

The notion of scalability was also particularly central to the EWG’s discussion on open and controlled access data. While open science is central to the HCA’s mission, in light of the international regulatory landscape, it quickly became apparent that achieving open access data (i.e., public data) globally would be a daunting task. Furthermore, a ‘one-size-fits-all’ approach to public data sharing could also inadvertently exclude certain regions, populations, or groups from contributing to the HCA because, for instance, of data protection regulatory requirements, ethical policy, or cultural sensitivities^12,45–47^. In response to this ongoing discussion and consultation of stakeholders within HCA leadership, the EWG integrated language throughout the toolkit (e.g., consent form templates, explanatory documents for ethics committees, material/data transfer agreements) to make the toolkit materials adaptable to the public release of data, while recognising that some datasets may require controlled (or managed) access. This approach allowed for immediately populating the HCA public database, while acknowledging that some contributors would need additional technical infrastructure to contribute their data responsibly.

Finally, a key element to ensure that contributors to the HCA and its users are implementing the available ethics resource is that documentation be readily actionable and implementable. Ethical and legal requirements can often seem distant from the realities of scientific research. Research, in turn, can sometimes seem overly technical or theoretical for the lay community. However, the success of an infrastructure initiative such as the HCA relies on building a common understanding between different stakeholders involved, including donors/research participants, communities, ethics committees, regulators, scientists, and funders^48^. In the context of the HCA, this meant, for instance, providing accessible explanation regarding the technology used (e.g., single-cell RNA sequencing, cellular profiling), but also, of concepts surrounding data sharing (open, controlled, etc.). The ethics toolkit was developed with this premise in mind, and in parallel to other HCA initiatives (public engagement, equity, diversity, and inclusion). For instance, language was carefully adapted to the proper audience (e.g., explanation to research ethics committees, consent documentation for donors/participants, explanation to regulators/lawyers, etc.).

In sum, the path to building an interoperable, scalable, and actionable ethics governance framework for the HCA involved assembling a multidisciplinary working group, supported by strong integration within the HCA technical and scientific communities. The efforts of the also closely align with those of other HCA initiatives, such as the HCA Equity Working Group, as these groups strive to ensure that the Atlas is built for the benefit of humanity^49^. Early commitment and support from HCA leadership were key to building a sound ethics governance framework, in synergy with the development of the Atlas, allowing for actionable implementation of foundational data sharing premises^22^ through resources for the scientific community contributing to the consortium. In time, however, we expect that further refinement, revision or adaption of the toolkit may be necessary, particularly as the data-sharing landscape evolves (for instance, due to technological changes, changes in the regulation of data protection, research ethics, use of artificial intelligence, etc.). This may require, for instance, refinement of consent wording, sharing agreements, or adaptation to data governance arrangements. This is a potential limitation of the current work, however, it may become an opportunity to further engage and consult active HCA contributors, developers and users.

Building on a number of key Open Science principles set forth by UNESCO in 2021, in order to develop a global public good benefitting humanity, careful consideration must be given to concepts such as: access, data, software and hardware, infrastructures, evaluation, educational resources, engagement of societal actors, and diversity of knowledge^24^. Doing so relies on the ability of the consortium members and stakeholders to operate within clear, implementable policy environments, to build sustainable and inclusive research infrastructures. It also calls for ongoing monitoring of emerging issues and an agile response to adapt the ethical framework to rapidly evolving social, scientific, and technological environments.
